# The Centriolar Satellite Protein AZI1 Interacts with BBS4 and Regulates Ciliary Trafficking of the BBSome

**DOI:** 10.1371/journal.pgen.1004083

**Published:** 2014-02-13

**Authors:** Xitiz Chamling, Seongjin Seo, Charles C. Searby, GunHee Kim, Diane C. Slusarski, Val C. Sheffield

**Affiliations:** 1 Department of Pediatrics, University of Iowa Interdisciplinary program of genetics, Iowa City, Iowa, United States of America; 2 Department of Ophthalmology and Visual Sciences, University of Iowa Carver College of Medicine, Iowa City, Iowa, United States of America; 3 Howard Hughes Medical Institute, Chevy Chase, Maryland, United States of America; 4 Department of Biology, University of Iowa, Iowa City, Iowa, United States of America; Washington University School of Medicine, United States of America

## Abstract

Bardet-Biedl syndrome (BBS) is a well-known ciliopathy with mutations reported in 18 different genes. Most of the protein products of the BBS genes localize at or near the primary cilium and the centrosome. Near the centrosome, BBS proteins interact with centriolar satellite proteins, and the BBSome (a complex of seven BBS proteins) is believed to play a role in transporting ciliary membrane proteins. However, the precise mechanism by which BBSome ciliary trafficking activity is regulated is not fully understood. Here, we show that a centriolar satellite protein, AZI1 (also known as CEP131), interacts with the BBSome and regulates BBSome ciliary trafficking activity. Furthermore, we show that AZI1 interacts with the BBSome through BBS4. AZI1 is not involved in BBSome assembly, but accumulation of the BBSome in cilia is enhanced upon AZI1 depletion. Under conditions in which the BBSome does not normally enter cilia, such as in BBS3 or BBS5 depleted cells, knock down of AZI1 with siRNA restores BBSome trafficking to cilia. Finally, we show that *azi1* knockdown in zebrafish embryos results in typical BBS phenotypes including Kupffer's vesicle abnormalities and melanosome transport delay. These findings associate AZI1 with the BBS pathway. Our findings provide further insight into the regulation of BBSome ciliary trafficking and identify AZI1 as a novel BBS candidate gene.

## Author Summary

Bardet-Biedl syndrome (BBS) is a genetically heterogeneous autosomal recessive ciliopathy with 18 causative genes reported to date. The syndrome is characterized by obesity, polydactyly, renal defects, hypogenitalism and retinal degeneration. Previous work has illustrated a role for BBS proteins in the trafficking of ciliary cargo proteins including MCHR1, SSTR3, and dopamine receptor 1. In addition, interaction of BBS proteins with other centriolar satellite proteins has been reported. In order to identify novel BBS interacting proteins and novel BBS candidate genes we generated a transgenic BBS4 mouse. In this study, we utilized the transgenic mice to identify a novel BBSome (a complex of eight BBS proteins) interacting protein, AZI1. We show that AZI1 physically binds to the BBSome via BBS4. We also suggest a negative role of AZI1 in ciliary trafficking of the BBSome: when AZI1 is depleted, more BBSome localizes to cilia. Using zebrafish as a model, we show that azi1 morphants are similar to bbs morphants, a finding that further implicates AZI1 with the BBS pathway. Our findings provide further insight into the regulation of BBSome ciliary trafficking and identify AZI1 as a BBS candidate gene.

## Introduction

Primary cilia are organized from centrioles that move to the cell periphery and form basal bodies. From the centrioles, microtubules extend and protrude from the cell surface to produce a cilium. Primary cilia house several signaling pathway receptors such as Hedgehog, Wnt and PDGFR, and are essential for tissue homeostasis, photoreceptor function, and olfaction [1], [2], [3]. Defective cilium formation leads to a shared set of phenotypes including retinal degeneration, polydactyly, situs inversus, hydrocephaly, and polycystic kidney disease, which are features of several pleiotropic genetic disorders including Alström syndrome (ALMS), Nephronophthisis (NPHP), Joubert Syndrome, and Bardet-Biedl syndrome (BBS) [4], [5], [6]. Many ciliary proteins form complexes and functional networks. For example, NPHP and MKS proteins form a modular complex at the transition zone that functions as a ciliary gate [7], [8], [9], [10], and intraflagellar transport (IFT) proteins form complexes involved in ciliary protein trafficking [11]. Similarly, seven BBS proteins and BBIP10 form a stable octameric complex, the BBSome [12], [13].

The BBSome localizes to both centriolar satellites and cilia and aberrant localization of several ciliary proteins including MCHR1, SSTR3, and dopamine receptor 1 has been observed in *Bbs2* and *Bbs4* null brain [14], [15]. In addition, interaction of BBS4 with other centriolar satellite proteins such as PCM1, and interaction of BBS9 with LZTFL1 have been reported [16], [17]. However, more BBSome cargoes and BBSome interacting proteins in mammalian cells and tissues remain to be identified, and the precise mechanisms by which BBSome trafficking activity is regulated remain to be determined.

In this study, we use a previously described transgenic Bbs4 mouse model [18] to identify additional BBSome interacting proteins. We report a centriolar satellite protein, AZI1, as a novel BBSome interacting protein, which physically binds with the BBSome via BBS4. BBS4 is a BBSome subunit known to localize to centriolar satellites, and the final subunit added during BBSome assembly [19]. It has been proposed that satellite proteins such as PCM1 interact with BBS4 prior to its incorporation into the BBSome [20]. Consistent with this hypothesis, we observed a separate centriolar satellite pool of BBS4 apart from the BBSome complex in HEK293T cells. Our results indicate that AZI1 is part of the PCM1-dependent centriolar complex containing BBS4. We also show that depletion of AZI1 does not affect BBSome complex formation, but decreases cilia formation and enhances trafficking of the BBSome to cilia. Finally, using zebrafish as a model, we show that *azi1* morphants are similar to *bbs* morphants, a finding that further implicates AZI1 with the BBS pathway, and makes *AZI1* a BBS candidate gene.

## Results

### Identification of AZI1 as a BBSome interacting protein

**Figure 1:**
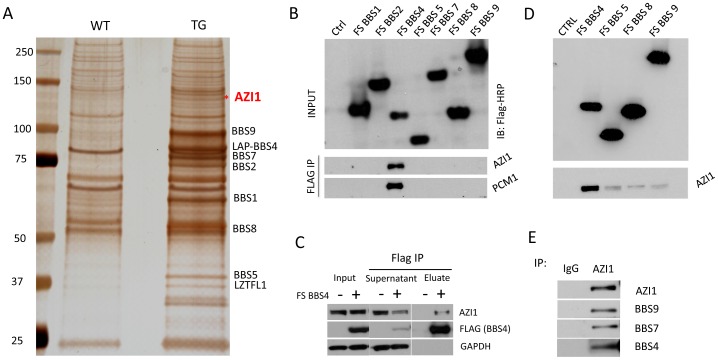
AZI1 interacts with the BBSome through BBS4. **A**) A silver stained gel from GFP IP sample of testis lysate. Lane 1 shows the wild-type (WT) precipitate and lane 2 is the precipitate of testis lysate from transgenic (TG) animals. The band labeled with an asterisk is the 120 KDa band identified as Azi1 by mass spectrometry. **B**) Western blot showing interaction of BBS4 with AZI1 and PCM1. FLAG and S tagged BBSome subunits were transfected in 293T cells and Co-IP was performed using FLAG agarose beads. The top blot shows efficient transfection of the BBS subunits detected by anti-FLAG antibody. The lower two blots were immunoblotted with AZI1 and PCM1 antibody, respectively. **C**) FLAG-BBS4 stable cell (293T) was co-immunoprecipitated with FLAG agarose beads. Input, supernatant and eluate were immunoblotted with, antibody against GAPDH to show that equal amounts were loaded, antibody against FLAG to show efficient precipitation of BBS4 by FLAG beads, and antibody against AZI1 to show amounts of AZI1 precipitated by BBS4 in our cell line. **D**) 293T cells stably expressing the FLAG tagged BBSome subunits BBS4, 5, 8, and 9 were co-IPed with FLAG agarose beads and immunoblotted with AZI1 antibody. Although all the subunits were able to precipitate AZI1, BBS4 showed the most efficient pull-down. **E**) Co-IP using AZI1 antibody was performed in 293T cells followed by Western blotting with different BBS antibody to show interaction of endogenous BBS proteins and AZI1.

To isolate BBSome interacting proteins in vivo, we generated a transgenic mouse line expressing LAP (localization and purification) tagged human *BBS4* gene [18]. Tissue lysates from transgenic mice were used for tandem affinity purification (TAP) in a manner similar to that previously described [17]. Although we could detect all BBSome components and Lztfl1 readily, we were not able to detect any other novel interacting proteins. We reasoned that most of the transiently interacting BBSome cargos and regulators are lost during the TAP procedure. Therefore, we decided to perform a single step immunoprecipitation (IP) to increase the chance of precipitating weakly interacting proteins. Whole tissue lysates from transgenic and wild type testis were used for IP. Using anti-GFP affinity beads, we pulled down all seven BBSome subunits from the transgenic sample (Fig. 1A); in addition, we were able to detect and identify a 120 KDa band as azacytidine-induced protein 1 (AZI1; also known as CEP131) (Fig. 1A, Fig. S1). Interestingly, Staples *et al.* [21] did not detect interaction of AZI1 with BBS4, which could be due to lack commercial BBS4 antibody that specifically detects BBS4.

*AZI1* maps to chromosome 17q25.3 in humans and the syntenic region of chromosome 11 in mouse. Eight different transcriptional variants of *AZI1* have been predicted in human, among which seven are predicted to produce proteins. Homology searches have shown that *AZI1* is highly conserved in taxa with a cilium or flagellum, particularly in organisms with canonical IFT and compartmentalized cilia [22].

### AZI1 interacts with BBS4 and other centriolar satellite proteins

To determine the AZI1 interacting subunit of the BBSome, 293T cells were transfected with individual BBSome subunits (with FLAG tags) and cell lysates were precipitated with FLAG affinity beads. AZI1 was specifically precipitated with BBS4 (Fig. 1B). We also compared the supernatants of FLAG-BBS4 expressing cells and 293T control cells. Western blotting with AZI1 antibody showed that about 50% of AZI1 is associated with BBS4 (Fig. 1C). Transiently transfected plasmids often fail to pull down weakly or indirectly interacting proteins; therefore, we performed additional IP experiments utilizing 293T cells stably expressing FLAG-tagged BBSome subunits (BBS4, BBS5, BBS8, and BBS9) (Fig. 1D). Using these stable cell lines, we were able to precipitate AZI1 with all individual BBSome subunits used, with the band resulting from BBS4 IP being the strongest compared to the other BBS proteins. These results indicate that AZI1 interacts with the BBSome through BBS4.

To confirm the interaction of AZI1 with the BBSome and to test its association with other centriolar satellite proteins, we generated 293T cells stably expressing FLAG tagged AZI1. Lysates from this stable cell line and parental cells (as a negative control) were subjected to co-IP with FLAG affinity beads, followed by Western blot analysis. Along with FS-AZI1, centriolar satellite proteins CEP290 and PCM1 were precipitated (Fig. S2A). In addition, all BBSome subunits tested (BBS4, BBS5, BBS7, BBS8, and BBS9) were also precipitated with AZI1. These results confirm that AZI1 is part of the centriolar satellite complex and interacts with the BBSome. Along with FS-AZI1, endogenous AZI1 was also precipitated suggesting that AZI1 forms a homo-oligomer (Fig. S2A). Interaction of endogenous AZI1 and BBSome proteins was verified by IP using AZI1 antibody in 293T cells (Fig. 1E).

**Figure 2:**
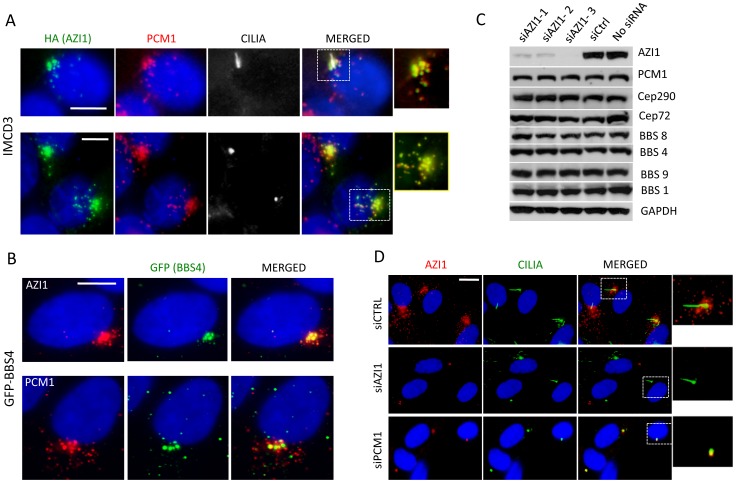
AZI1 co-localizes with BBS4 to the centriolar satellite. **A**) AZI1 and PCM1 co-localize at the centrosome. IMCD3 cells were transfected with HA-AZI1 construct and stained with antibody against HA (green) and PCM1 (red). AZI1 and PCM1 co-localize at the centrosome, in the presence (row 1) as well as absence of cilia (row 2). γ-tubulin and acetylated α-tubulin staining were used to identify the basal body and cilia. **B**) BBS4 co-localizes with AZI1, which resembles its co-localization with PCM1. RPE-1 cells stably expressing GFP-BBS4 were used; anti-GFP antibody was used to detect BBS4 (green); anti-AZI1 (row 1) or anti-PCM1 (row 2) antibody is shown in red. **C**) Western blotting shows expression levels of BBS and other satellite proteins upon AZI1 knockdown. Three different siRNAs were used, and efficient and specific knockdown of AZI1 by all three siRNAs are shown. Antibody against various BBS and satellite proteins were used to show that loss of AZI1 does not cause significant differences in the expression of those proteins. GAPDH is used as a loading control. **D**) Centriolar satellite localization of AZI1 (red) is confirmed in RPE-1 cells. siRNA based depletion diminished AZI1 from centriolar satellites. Depletion of PCM1 also depletes AZI from satellites but AZI1 at core centriolar areas remain intact. Insets show enlarged view of the centrosomal region. Green is γ-tubulin and acetylated α-tubulin staining for basal body and cilia, respectively. Scale bar, 10 µm.

We next examined whether AZI1 and the BBSome co-localize. Previously, we showed that a subset of GFP-BBS4 and endogenous BBS9 localize to the centriolar satellites in RPE1 cells [12], [17]. Here, we show that AZI1 co-localizes with BBS4 in our GFP-BBS4 expressing cells (Fig. 2B). We transfected HA-tagged AZI1 construct in IMCD3 cells and found that PCM1 also co-localizes with AZI1 in ciliated as well as non-ciliated cells (Fig. 2A); results from 293T cells stably expressing FS-AZI1 or FS-PCM1 show similar co-localization (Fig. S2B).

### Satellite protein expression and localization is independent of AZI1 expression

Since AZI1 interacts with the BBSome and satellite proteins, we wanted to evaluate the effect of AZI1 depletion on those proteins. First, we tested the efficacy of our *AZI1* siRNAs by Western blot analysis (Fig. 2C) and by immunofluorescence microscopy (Fig. 2D); significant loss of AZI1 by each siRNA used was observed with both methods. Then we performed Western blot analysis on AZI1 depleted cells using antibody against various satellite and BBSome proteins. Our result shows that *AZI1* depletion does not affect expression of any BBSome or satellite proteins (Fig. 2C). Furthermore, *AZI1* depletion did not change the centrosomal localization of CEP290 or PCM1 (Fig. S2C). We then depleted PCM1 and CEP290 and analyzed AZI1 localization in RPE-1 cells. Unlike non-ciliated U2O6 cells [21], *CEP290* knockdown in our ciliated RPE-1 cells did not show significant change in centriolar localization of AZI1 (Fig. S4B); however, similar to previously reported data [21], [23], loss of AZI1 from the centriolar satellite was observed upon depletion of PCM1 (Fig. 2D). Interestingly, a pool of AZI1 at the core centriolar region remains intact despite PCM1 knockdown (inset Fig. 2D). The results indicate that the satellite pool but not the core centriolar localization of AZI1 is dependent on PCM1.

### BBS4 forms a separate centriolar satellite complex with PCM1 and AZI1

**Figure 3:**
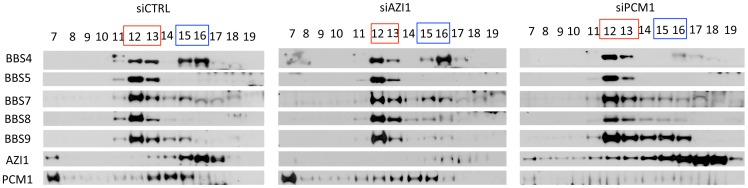
BBS4 is part of a PCM1 dependent centriolar satellite complex. 293T cells were transfected with siRNA against CTRL, AZI1, and PCM1, and the cell lysates were separated by 10–40% sucrose gradient centrifugation and fractionation. Thirteen fractions, (from 7–19, 19 being the heaviest fraction) were run on an SDS-PAGE gel and analyzed by immunoblotting with antibodies against various BBS proteins, AZI1, and PCM1. Fractions marked red represent the peak for the BBSome. Fractions marked blue is the peak for centriolar satellite pool of BBS4, along with centrosomal proteins, AZI1 and PCM1.

Since AZI1 interacts with BBS4, we posited that AZI1 would fractionate with BBS4 in a sucrose gradient. Therefore, we performed sucrose gradient ultracentrifugation and fractionation of 293T cell lysates followed by SDS-PAGE. Immunoblotting of the fractions with different BBS proteins, PCM1 and AZI1 antibodies revealed that BBS4 is fractionated in two distinct complexes: one with the BBSome subunits (fractions 12–13) and the other with PCM1 and AZI1 (fractions 15–16) (Fig. 3). BBS4 has been shown to be a satellite protein, which interacts with other large centriolar satellite proteins including PCM1 and Cep290 [16], [20], [24]. These findings and our results suggest that BBS4 is part of a satellite complex prior to its incorporation into the BBSome complex. Of note, such separation of BBS4 is not readily observed in testis lysates (Fig. S3) or in RPE-1 cells [12], [17], but is observed in RPE-1 cells expressing LAP-BBS4 (Fig. S3). It is likely that the centriolar pool of BBS4 is more evident when high amounts of BBS4 is free from the BBSome complex, such as in cells overexpressing BBS4 (LAP-BBS4) or in mostly proliferating, non-ciliated cells (293T).

To further explore whether the second pool of BBS4 is in fact a separate centriolar satellite pool, we knocked down PCM1 in 293T cells. Since PCM1 is essential for holding centriolar satellite together [21], [25], [26], [27], depletion of PCM1 diminished BBS4 from the centriolar satellite pool (fractions 15–16), but BBS4 in the BBSome (fraction 12–13) remained intact (Fig. 3). In contrast, knockdown of AZI1 did not alter the amount of BBS4 found in the BBSome or in the centriolar complex: BBS4 was detected in fractions 12–13 as well as 15–16. Furthermore, no significant change in the fractionation of PCM1 was observed in AZI1 depleted samples. Similar to control knockdown, PCM1 was observed in fraction 7 and 13–15 in AZI1 depleted cells. These results indicate that the centriolar satellite complex pool of BBS4 is dependent on the presence of PCM1 but not AZI1, and that the absence of either PCM1 or AZI1 does not affect BBSome formation.

### AZI1 is a negative regulator of BBSome ciliary trafficking

**Figure 4:**
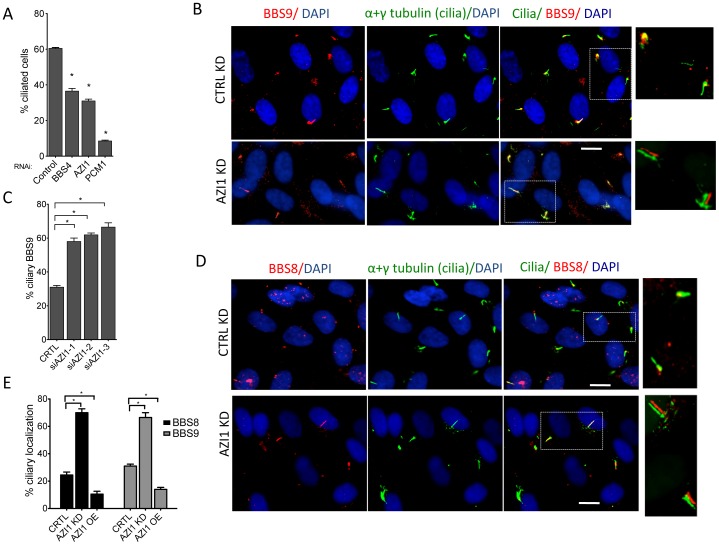
AZI1 knockdown reduces ciliogenesis but increases ciliary localization of the BBSome. **A**) RPE-1 cells were transfected with siRNA against *BBS4*, *PCM1*, and *AZI1*. Approximately 500 cells per sample were counted. **B**) AZI1 depletion increases the ciliary localization of BBS9 compared to the control knockdown. Red staining represents BBS9, and cilia are stained with acetylated α-tubulin. Nuclei are stained blue with DAPI. **C**) Graph showing a significant increase in cells with ciliary BBS9 upon AZI1 knockdown by different siRNAs. **D**) BBS8 (red) is used as a BBSome marker to confirm increased ciliary localization of the BBSome upon AZI1 knockdown. Cilia (green) in the insets of figures **B** and **D** are slightly shifted to show ciliary localization of BBS proteins. **E**) The graph shows a significant increase in the number of ciliated cells with BBS8 or BBS9 upon AZI1 knockdown, and decrease in ciliary BBS8 or BBS9 localization upon AZI1 overexpression. Approximately 250 ciliated cells were counted in control as well as AZI1 knockdown culture of RPE-1 cells. All data are presented in mean +/− SEM. Significance is calculated using Student's t-test for C and E, and one way ANOVA for A. P<0.05 is considered significant for each analysis.

**Figure 5:**
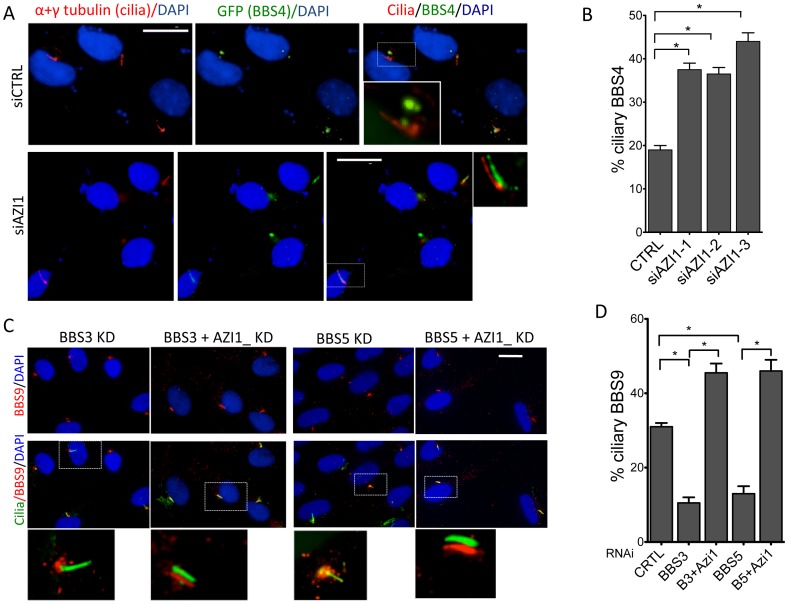
AZI1 knockdown increases ciliary localization of BBS4. **A**) RPE-1 cells expressing GFP-BBS4 were depleted of AZI1, and the number of cells with ciliary GFP (BBS4) was counted. BBS4 is stained with GFP (green), Acetylated α-tubulin was used to detect cilia (red). **B**) Graph showing significant increases in ciliary localization of BBS4 upon AZI1 knockdown. **C**) Depletion of AZI1 in BBS3 and BBS5 depleted cells restores ciliary BBSome localization. RPE-1 cells were transfected with siRNA as indicated, and BBS9 (red) localization was analyzed. Cilia (green) in the insets of figures **A** and **C** are slightly shifted to show ciliary localization of BBS proteins. **D**) Cilia containing BBS9 at different conditions were counted and presented graphically. All data are presented in mean +/− SEM. Significance is calculated using the Student's t-test for C and E, and one way ANOVA for A. P<0.05 is considered significant for each analysis.

Since AZI1 interacts with the BBSome, but BBSome assembly is not dependent on AZI1, we investigated the role of AZI1 in ciliary trafficking of the BBSome. First, we knocked down AZI1 using siRNAs in RPE-1 cells. We found that in contrast to blocked ciliogenesis upon knockdown of PCM1, depletion of AZI1 reduced cilia formation by approximately 50% (Fig. 4A). Second, we tested whether depletion of BBSome subunits has any effect on AZI1 localization and vice versa. siRNA-based knockdown of BBS genes had no effect on the centriolar satellite localization of AZI1 (Fig. S4). However, ablation of *AZI1* expression altered the ciliary localization of the BBSome based on using BBS8, BBS9 and GFP-BBS4 as markers for the BBSome (Fig. 4B–E, 5A–B, S5). In control siRNA transfected RPE1 cells, 35% of the ciliated cells demonstrate ciliary localization of BBS9. *AZI1* knockdown by three different siRNAs showed a significant increase of ciliary BBS9 to 58%, 60% or 65% (Fig. 4C, 4E, S5A). Similarly, BBS8 ciliary localization was increased to 70% upon AZI1 knockdown compared to 28% ciliary BBS8 in control cells (Fig. 4D–E). We also assessed the effect of *AZI1* depletion on BBS4 by using RPE1 cells stably expressing GFP-BBS4. Approximately 20% of the ciliated cells contain ciliary GFP-BBS4 (Fig. 5A–B) in our stable cells. Upon *AZI1* knockdown, ciliary localization of BBS4 increases to 38%, 35% or 44% respectively (Fig. 5A, 5B S5B). Conversely, overexpression of AZI1 significantly reduced ciliary localization of BBS8 and BBS9 (Fig. 5E and Fig. S6). These results indicate that the absence of AZI1 enhances ciliary localization of the entire BBSome, and implicates AZI1 as a negative regulator of ciliary BBSome trafficking.

### Depletion of AZI1 restores BBSome trafficking to cilia in BBS3 and BBS5 depleted cells

Knockdown of LZTFL1, a BBSome interacting protein known to negatively regulate ciliary trafficking of the BBSome, has been shown to restore ciliary BBS8 and BBS9 in BBS3 and BBS5 depleted cells, which normally lack the BBSome proteins in cilia [17]. Since AZI1 also negatively regulates ciliary BBSome localization, we tested whether *AZI1* knockdown has a similar effect. When BBS genes are knocked down, no BBS9 is seen in cilia with BBS9 accumulating at the centrosome or the ciliary base (Figs. 5C and S7). In contrast, siRNA knockdown of AZI1 in BBS3 or BBS5 depleted cells restores the ciliary localization of BBS9 (Figs. 5C–D). Although not as evident as in BBS3 and BBS5 depleted cells, similar rescue was observed in BBS2 and BBS8 depleted cells (Fig. S7). As seen with LZTFL1, knockdown of AZI1 in BBS1 depleted cells could not rescue BBS9 localization to cilia (Fig. S7); most BBS9 in those cells clustered around the centrosome.

### AZI1 is involved in BBS dependent functions in zebrafish

**Figure 6:**
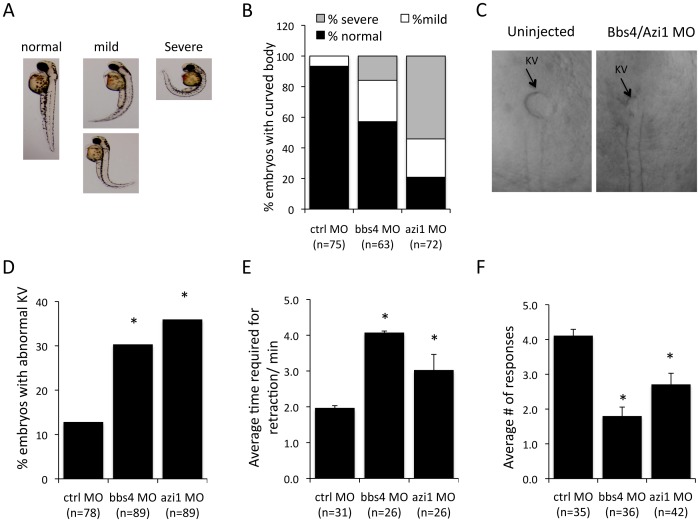
Azi1 (Cep131) knockdown causes defects in KV formation, melanosome trafficking and vision defects in zebrafish. Embryos were untreated or injected with 125 µM Bbs4 MO or 150 µM cep131 MO as previously described [28], [29], [31], [33]. **A**) *azi1*, as well as *bbs4* morpholino injection causes a mild to severe body curvature phenotype in the 48 hpf larva. Images indicate examples of normal, mild or severe phenotypes. **B**) Quantification of body curvature. More of the *azi1* morphants have severe body curvature than *bbs4* morphants. **C**) Micrographs show KVs from MO injected and uninjected 8–10 somite stage embryos. **D**) *bbs4* MO caused KV abnormalities in 30% of embryos, and *azi1* MO caused 35% abnormal KV compared with 12% in control embryos (P<0.001 Fisher's exact test). **E**) *bbs4* MO and *azi1* MO delayed melanosome retrograde transport to 4.04+/−0.43 min, and 3.03+/−0.12 min, respectively compared to the control MO, in which the complete transport occurs within 1.92+/−0.04 minutes. **F**) Vision assays were performed on the morphants by observing their response to dark/light cues as previously described [33], [35]. For each fish the vision response tested 5 times. For C and D data are presented mean +/− SEM, P<0.01 by one-way ANOVA with Tukey post-test. The number of embryos used is presented in the figure.

Knockdown of BBS genes in zebrafish using morpholino oligonucleotides (MO) results in reduced Kupffer's vesicle (KV) and cilia formation ([28], [29], [30], [31]. It has also been demonstrated that *azi1* knockdown in zebrafish resembles BBS morphants including shortened and scarce cilia in Kupffer's vesicles and randomized left-right symmetry [32]. Abnormal body curvature was also significantly increased in *azi1* morphants (Figs. 6A–B). To distinguish the *azi1*-induced defects from general cilia function vs. BBS-related defects, we tested the impact of *azi1* knockdown on BBS-associated phenotypes including melanosome transport and visual function [31], [33], [34].

Similar to *bbs4* morphants, *azi1* knockdown delays retrograde melanosome transport, causes vision defects as well as abnormalities in KV morphogenesis in zebrafish embryos (Figs. 6C–F). *azi1* knockdown resulted in approximately 35% of zebrafish embryos having KV abnormalities, which is similar to the 30% abnormal KV observed in *bbs4* MO treated embryos, and significantly different compared with the 12% abnormal KV observed in control morphant embryos (Fig. 6D). Another cardinal feature of BBS knockdown is delayed melanosome trafficking [28]. Similar to *bbs4* knockdown, *azi1* morphants demonstrate delayed retrograde trafficking of melanosomes compared to controls (Fig. 6E). In addition, vision startle response [35] was assessed in *azi1* morphants by observing their responses to dark/light cues. For each fish the response was tested 5 times. On average, control MO treated fish respond to the dark/light cue, 4.2 out of 5 times; *bbs4* and *azi1* morphants respond 2 and 2.6 times, respectively (Fig. 6F), indicating that the *bbs4* and *azi1* deficient fish are visually impaired. Our data clearly indicate BBS-associated functions of azi1 in zebrafish.

## Discussion

Recent work on AZI1 (CEP131) has implicated its role in cilia formation, genome stability, and tumor formation [21], [32], [36], [37]. Interaction of AZI1 with centriolar satellite proteins PCM1 and CEP290, as well as with microtubule motor protein p150, has been reported [21]. In the centrosome, PCM1 and CEP290 interact with each other, as well as with BBS4, which is also known to interact with p150 [16]. In this study, we identify and report AZI1 as a BBS4 interacting protein through which it interacts with the BBSome. We report that AZI1 interacts with PCM1 and BBS4, and negatively regulates trafficking of the BBSome to cilia. Upon PCM1 depletion, the localization of AZI1 to centriolar satellites is lost but its localization at the centriole is maintained with the help of pericentrin and CEP290 [21]. Localization of BBS4 to centriolar satellites is also dependent on PCM1 (Fig. 3); therefore, PCM1 acts as a scaffold upon which these satellite proteins interact. Interactions observed among AZI1, BBS4, PCM1, and CEP290 suggests that they are part of the same complex.

Interaction of BBS4 with satellite proteins and intrinsic BBS4 satellite localization independent of other BBSome proteins [19] indicate the possibility of a centriolar satellite complex containing BBS4, apart from the BBSome complex. We report such a PCM1-dependent satellite complex consisting of BBS4 in 293T and GFP-BBS4 cells. Lack of such a distinct complex in normal ciliated RPE cells indicates that the distinct centriolar pool of BBS4 is more evident when there is an excess of BBS4 or absence of primary cilia. It is likely that BBS4 has a higher affinity for the BBSome complex than it does for the satellite complex. In cells overexpressing BBS4, excess BBS4 cannot be incorporated into the BBSome complex, and in non-ciliated cells (such as proliferating 293T cells) BBSome complex formation may not be as robust as in ciliated cells. As a result, more BBS4 remains as a part of the centriolar satellite complex in those cells.

The proteins in the centriolar satellite appear to have a dual role: Recruiting proteins involved in cilia assembly to a location near the ciliary base, and controlling the entry of recruited proteins into the cilium. PCM1 functions as a scaffold protein to recruit centriolar satellite proteins and is essential for centriolar satellite formation and cilia assembly. Absence of PCM1 results in complete dispersal of satellite and BBS proteins [21], [25], [26], [27], and as a consequence the failure to form cilia or loss of cilia. AZI1, which also localizes to centriolar satellites, appears to restrict ciliary recruitment of the BBSome. Of note, AZI1 is not required to maintain the integrity of the centriolar complex, and the centriolar pool of BBS4 remains intact even in the absence of AZI1. Our results indicate that AZI1 is not involved in BBSome formation but it negatively affects ciliary recruitment of the BBSome.

**Figure 7:**
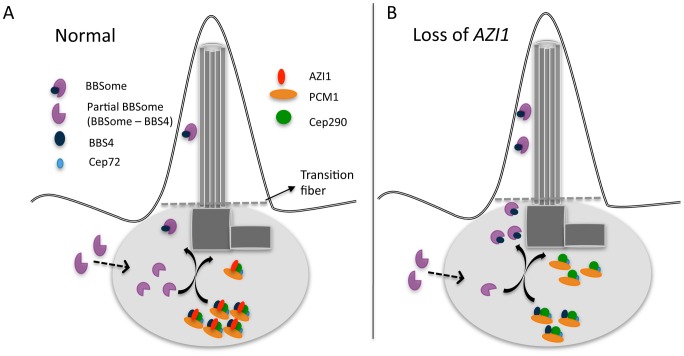
A model showing centrosomal complex of BBS4, BBSome complex formation, and its ciliary localization. **A**) 1. Partial BBSome complex (without BBS4) arrives near the centrosome, where BBS4 is part of a satellite complex. At the centrosome BBS4 is incorporated into the BBSome and a stable holo-BBSome complex is formed, which is trafficked to cilia while the rest of the satellite complex remain at the centrosome. A potential rate-limiting factor of BBSome entry into cilia is availability of the BBSome complex that can enter cilia. **B**) Loss of AZI1 weakens the interaction of BBS4 with the satellite complex, and more BBSome complex is available for entry into cilia.

Similar to PCM1, AZI1 binds to BBS4 and may play a role in sequestering BBS4 and limiting its availability for incorporation into the BBSome [20]. Therefore, one potential rate-limiting factor related to BBSome entry into cilia is the limited availability of the holo-BBSome that can enter cilia. It is likely that PCM1, with its binding role at the satellite, holds and sequesters BBS4 within the satellite complex with the assistance of AZI1. We showed previously that a partial BBSome complex (without BBS4) arrives at the centrosome to incorporate BBS4 as its last subunit [19] (Fig. 7). Upon the addition of BBS4 to the BBSome, the holo-BBSome can enter cilia while other satellite proteins remain at the ciliary base [17] (Fig. 7A). When AZI1 is depleted, more BBS4 can associate with the BBSome and more holo-BBSome is available for ciliary entry (Fig. 7B).

It should be noted that AZI1 interacts with BBS4 in the satellite complex, as well as with BBS4 when it is part of the complete BBSome (Fig. S2A). One possibility is that AZI1 transiently associates with the BBSome during transfer of BBS4 to the BBSome to complete the complex. However, we cannot rule out the possibility that AZI1 may directly regulate BBSome ciliary trafficking through phosphorylation or similar post translation modification events. Determination of the signals that regulate BBSome binding to AZI1 and its release for ciliary recruitment is an interesting area for future work. In addition, further study is essential to understand the precise mechanism by which AZI1 regulates BBSome ciliary entry.

The negative regulation of ciliary BBSome trafficking is also a characteristic of LZTFL1, a recently identified protein that interacts with the BBSome [17]. The recent discovery of an *LZTFL1* mutation in a BBS patient [38] suggests that identifying new interacting proteins assists in discovery of novel disease causing genes. Our zebrafish data show that morpholino knockdown of *azi1*, similar to *bbs* gene knockdown, causes BBS-like phenotypes in zebrafish, and confirms the association of AZI1 with the BBS pathway in an animal model.

## Methods

### Ethics statement

The University Animal Care and Use Committee of the University of Iowa approved all animal work in this study (animal protocol number: 1003062). Every person involved in handling mice was properly trained to the standards established by the committee.

### Transgenic (LAP-BBS4) mice

LAP-BBS4 transgenic mice were described previously [18]. Genotypes for the *lap-BBS4* transgene were determined using the following primers: GTCCTGCTGGAGTTCGTGAC and GGCGAAATATCAATGCTTGG).

### Antibodies, plasmids, and reagents

BBS4 antibody was kindly provided by Dr. Maxence Nachury (Stanford University). Antibodies against AZI1 (HPA024019), BBS9 (HPA021289), BBS8 (HPA003310), anti-acetylated tubulin (6-11B-1), and FLAG affinity beads (A2220) were purchased from Sigma (St. Louis, MO, USA); Antibodies against BBS7 (8961-1-AP) and BBS5 (5691-1-AP) were purchased from ProteinTech Group; antibody against PCM1 (A301-149A) from Bethyl Lab (Montgomery, TX, USA); GFP antibody (A11120), Alexa 488-conjugated, and Alexa 568-conjugated secondary antibodies were purchased from Invitrogen (Carlsbad, CA, USA). S-agarose beads for immunoprecipitation were purchased from EMD/Millipore (San Diego, CA).

Expression vectors for BBS genes were described previously [30]. *AZI1* was PCR amplified from a fetus cDNA library and cloned into CS2 plasmids with HA and FS (FLAG and S) tags. All small interfering RNAs (siRNAs) were purchased from Dharmacon (ON-TARGETplus SMARTpool). siRNA against CEP290 was SMARTpool (L-014590-00-0005). For *AZI1*, individual ON-TARGETplus siRNA were purchased; J-02335-05 (AGAUUGAGCUGGUCAUUCA), J-02335-08 (CAACGGAGGCCCACAGACUU), J-02335-07 (CCUGAACGUCCUGGAUGA), and L-02335-00-0005 (SMARTPpool). All siRNAs were transfected at 50 nM concentrations with RNAiMAX (Invitrogen) following the manufacturer's protocol.

### Co-immunoprecipitation (Co-IP) and immunofluorescence

Mice were sacrificed by cervical dislocation following IACUC guidelines and tissues were harvested for protein extraction. Co-IP on testis lysate was performed following the protocol described previously [12], [17], [39] and the purified proteins were separated in 4–12% NuPAGE gels (Invitrogen), and visualized with SilverQuest Silver Staining Kit (Invitrogen). Excised gel slices were submitted to the University of Iowa Proteomics Facility and proteins were identified by mass spectrometry using LC-MS/MS.

Co-IP on the 293T cells and the cells stably expressing FLAG-AZI1 were performed following the previously described protocol [17], [30]. For immunofluorescence, hTERT-RPE1 and 293T cells were seeded on glass cover slips in 24-well plates and maintained in DMEM/F12 or DMEM media (Invitrogen) supplemented with 10% FBS. siRNA transfection, cell fixation, staining and microscopy were performed as described previously [17]. Cilia were counted after staining with acetylated α-tubulin and γ-tubulin to mark cilia and the basal body.

### Quantitative real-time PCR and sucrose gradient ultracentrifugation

For gene knockdown, cells were transfected with siRNAs using RNAiMAX for 48 hrs. qPCR and/or Western blot analysis was used to confirm gene knockdown. RNA was extracted using IBI RNA extraction kit (cat# IBI47302) following the manufacturer's protocol and qPCR was performed as described previously [18].

For sucrose gradients, protein extract from one 10-cm dish of 293T cells transfected with PCM1, AZI1 or control siRNA was lysed and concentrated with a Microcon Centrifugal Filter Device (50,000 MWCO; Millipore). Sucrose gradient, TCA/acetone precipitation, SDSPAGE, and immunoblotting were performed as described previously [17].

### Analysis of Kupffer's vesicle, melanosome transport assay, and vision startle response assay

All assays were performed as published [28], [33] using MOs against the translation start site of *azi1* (MO- ATGGACTGCGGGTTGTATGCATCTT) and *bbs4* [28].

### Data analysis

Analysis of multiple groups was performed by one-way ANOVA, Student's t-test, Fisher's exact test or TUKEY as indicated. Error bars indicate SEM unless otherwise indicated.

## Supporting Information

Figure S1 Identification of AZI1. Screen-shot from scaffold3 software showing the unique peptides identified from the 120 KD band. The peptide coverage (**A**) and mass spectrum plot (**B**) shows identification of the AZI1.
(TIF)

Figure S2 AZI1 interacts with the BBSome complex and satellite proteins. **A**) FLAG and S tagged AZI1 were stably expressed in 293T cells, and Co-IP was performed using FLAG agarose beads. Input (total lysate) and the final precipitate were run on SDS-PAGE and Western blotted using antibody against CEP290, PCM1, BBS4, BBS5, BBS7, BBS8, and BBS9. Centriolar proteins as well as the other BBSome subunits along with FS-AZI1, and endogenous AZI1 were precipitated by AZI1. **B**) AZI1 co-localizes with PCM1 in non-ciliated cells. 293T cells stably expressing FS-PCM1 or FS-AZI1 were stained with antibody against S-tag (red) and PCM1 or AZI1 (green). **C**) siRNA knockdown of AZI1 has no effect on centriolar satellite localization of PCM1 or Cep290 (red). Cilia are stained with acetylated α-tubulin. Nuclei are stained blue with DAPI.
(TIF)

Figure S3 BBS4 is part of a centriolar satellite complex. Sucrose gradient (5%–25%) centrifugation followed by Western blotting was performed on protein lysates from testis and LAP-BBS4 stable cells. Two pools of BBS4 are seen in GFP-BBS4 cells. Although BBS4 extends towards the heaver fraction where PCM1 is seen, no evident separate pools of BBS4 was seen in the testis.
(TIF)

Figure S4 Localization of AZI1 is not affected by *BBS* knockdown. RPE-1 cells were transfected with siRNA against different BBS genes and the localization of AZI1 was analyzed. **A**) Localization of AZI1 upon control knockdown. **B**) AZI1 localization upon different BBSome proteins and Cep290 knockdown. No significant difference in the centriolar satellite localization of AZI1 is detected upon BBS proteins or Cep290 knockdown. Cilia and basal body are stained with acetylated α-tubulin and γ-tubulin respectively; red staining is AZI1, and nuclei are stained (blue) with DAPI.
(TIF)

Figure S5 AZI1 knockdown effects the ciliary localization of the BBSome. Ciliary localization of BBS9 (red) (**A**) or GFP-BBS4 (green) (**B**) is increased upon AZI1 knockdown by various *AZI1* siRNAs. Cilia (green) in figure **A** is slightly shifted to show BBS9 localizes in cilia. Cilia are stained with acetylated α-tubulin. Nuclei are stained blue with DAPI.
(TIF)

Figure S6 Overexpression of AZI1 reduces ciliary localization of BBS9. Cells were transfected with 0.25 µg of HA-AZI1 construct, and ciliary localization of BBS9 was analyzed. **A**) In control cells, ciliary BBS9 (red) is apparent, but no ciliary localization of BBS 9 in the AZI1 overexpressed cells was observed **B**). Effective transfection and HA-AZI1 expression is indicated in the last two images; HA staining (pseudo colored green) is also included for better comparison. Cilia are stained with acetylated α-tubulin and nuclei are stained with DAPI.
(TIF)

Figure S7 Localization of BBS9 upon knockdown of BBS proteins and AZI1. BBS proteins (BBS1, BBS2, and BBS8) are depleted in RPE-1 cells and loss of BBS9 (red) localization in cilia (green) is apparent (first row each panel). Knockdown of AZI1 in cells depleted of BBS protein rescues ciliary localization of BBS9 except in BBS1 depleted cells (second row each panel).
(TIF)

## References

[1] Singla V, Reiter JF (2006) The primary cilium as the cell's antenna: signaling at a sensory organelle. Science 313: 629–633. 16888132 10.1126/science.1124534

[2] Goetz SC, Anderson KV (2010) The primary cilium: a signalling centre during vertebrate development. Nat Rev Genet 11: 331–344. 20395968 10.1038/nrg2774 PMC3121168

[3] Williams CL, Li C, Kida K, Inglis PN, Mohan S, et al (2011) MKS and NPHP modules cooperate to establish basal body/transition zone membrane associations and ciliary gate function during ciliogenesis. J Cell Biol 192: 1023–1041. 21422230 10.1083/jcb.201012116 PMC3063147

[4] Badano JL, Katsanis N (2006) Life without centrioles: cilia in the spotlight. Cell 125: 1228–1230. 16814708 10.1016/j.cell.2006.06.013

[5] Hildebrandt F, Zhou W (2007) Nephronophthisis-associated ciliopathies. J Am Soc Nephrol 18: 1855–1871. 17513324 10.1681/ASN.2006121344

[6] Zariwala MA, Knowles MR, Omran H (2007) Genetic defects in ciliary structure and function. Annu Rev Physiol 69: 423–450. 17059358 10.1146/annurev.physiol.69.040705.141301

[7] Chih B, Liu P, Chinn Y, Chalouni C, Komuves LG, et al (2012) A ciliopathy complex at the transition zone protects the cilia as a privileged membrane domain. Nat Cell Biol 14: 61–72. 22179047 10.1038/ncb2410

[8] Craige B, Tsao CC, Diener DR, Hou Y, Lechtreck KF, et al (2010) CEP290 tethers flagellar transition zone microtubules to the membrane and regulates flagellar protein content. J Cell Biol 190: 927–940. 20819941 10.1083/jcb.201006105 PMC2935561

[9] Garcia-Gonzalo FR, Corbit KC, Sirerol-Piquer MS, Ramaswami G, Otto EA, et al (2011) A transition zone complex regulates mammalian ciliogenesis and ciliary membrane composition. Nat Genet 43: 776–784. 21725307 10.1038/ng.891 PMC3145011

[10] Sang L, Miller JJ, Corbit KC, Giles RH, Brauer MJ, et al (2011) Mapping the NPHP-JBTS-MKS protein network reveals ciliopathy disease genes and pathways. Cell 145: 513–528. 21565611 10.1016/j.cell.2011.04.019 PMC3383065

[11] Pedersen LB, Rosenbaum JL (2008) Intraflagellar transport (IFT) role in ciliary assembly, resorption and signalling. Curr Top Dev Biol 85: 23–61. 19147001 10.1016/S0070-2153(08)00802-8

[12] Nachury MV, Loktev AV, Zhang Q, Westlake CJ, Peranen J, et al (2007) A core complex of BBS proteins cooperates with the GTPase Rab8 to promote ciliary membrane biogenesis. Cell 129: 1201–1213. 17574030 10.1016/j.cell.2007.03.053

[13] Loktev AV, Zhang Q, Beck JS, Searby CC, Scheetz TE, et al (2008) A BBSome subunit links ciliogenesis, microtubule stability, and acetylation. Dev Cell 15: 854–865. 19081074 10.1016/j.devcel.2008.11.001

[14] Domire JS, Green JA, Lee KG, Johnson AD, Askwith CC, et al (2011) Dopamine receptor 1 localizes to neuronal cilia in a dynamic process that requires the Bardet-Biedl syndrome proteins. Cell Mol Life Sci 68: 2951–2960. 21152952 10.1007/s00018-010-0603-4 PMC3368249

[15] Berbari NF, Lewis JS, Bishop GA, Askwith CC, Mykytyn K (2008) Bardet-Biedl syndrome proteins are required for the localization of G protein-coupled receptors to primary cilia. Proc Natl Acad Sci U S A 105: 4242–4246. 18334641 10.1073/pnas.0711027105 PMC2393805

[16] Kim JC, Badano JL, Sibold S, Esmail MA, Hill J, et al (2004) The Bardet-Biedl protein BBS4 targets cargo to the pericentriolar region and is required for microtubule anchoring and cell cycle progression. Nat Genet 36: 462–470. 15107855 10.1038/ng1352

[17] Seo S, Zhang Q, Bugge K, Breslow DK, Searby CC, et al (2011) A novel protein LZTFL1 regulates ciliary trafficking of the BBSome and Smoothened. PLoS Genet 7: e1002358. 22072986 10.1371/journal.pgen.1002358 PMC3207910

[18] Chamling X, Seo S, Bugge K, Searby C, Guo DF, et al (2013) Ectopic Expression of Human BBS4 Can Rescue Bardet-Biedl Syndrome Phenotypes in Bbs4 Null Mice. PLoS One 8: e59101. 23554981 10.1371/journal.pone.0059101 PMC3598656

[19] Zhang Q, Yu D, Seo S, Stone EM, Sheffield VC (2012) Intrinsic protein-protein interaction-mediated and chaperonin-assisted sequential assembly of stable bardet-biedl syndrome protein complex, the BBSome. J Biol Chem 287: 20625–20635. 22500027 10.1074/jbc.M112.341487 PMC3370246

[20] Stowe TR, Wilkinson CJ, Iqbal A, Stearns T (2012) The centriolar satellite proteins Cep72 and Cep290 interact and are required for recruitment of BBS proteins to the cilium. Mol Biol Cell 23: 3322–35. 22767577 10.1091/mbc.E12-02-0134 PMC3431927

[21] Staples CJ, Myers KN, Beveridge RD, Patil AA, Lee AJ, et al (2012) The centriolar satellite protein Cep131 is important for genome stability. J Cell Sci 125: 4770–4779. 22797915 10.1242/jcs.104059

[22] Gabernet-Castello C, Dubois KN, Nimmo C, Field MC (2011) Rab11 function in Trypanosoma brucei: identification of conserved and novel interaction partners. Eukaryot Cell 10: 1082–1094. 21642507 10.1128/EC.05098-11 PMC3165442

[23] Akimov V, Rigbolt KT, Nielsen MM, Blagoev B (2011) Characterization of ubiquitination dependent dynamics in growth factor receptor signaling by quantitative proteomics. Mol Biosyst 7: 3223–3233. 21956701 10.1039/c1mb05185g

[24] Jin H, White SR, Shida T, Schulz S, Aguiar M, et al (2010) The conserved Bardet-Biedl syndrome proteins assemble a coat that traffics membrane proteins to cilia. Cell 141: 1208–1219. 20603001 10.1016/j.cell.2010.05.015 PMC2898735

[25] Ge X, Frank CL, Calderon de Anda F, Tsai LH (2010) Hook3 interacts with PCM1 to regulate pericentriolar material assembly and the timing of neurogenesis. Neuron 65: 191–203. 20152126 10.1016/j.neuron.2010.01.011 PMC2902371

[26] Lee JY, Stearns T (2013) FOP is a centriolar satellite protein involved in ciliogenesis. PLoS One 8: e58589. 23554904 10.1371/journal.pone.0058589 PMC3595297

[27] Wang G, Chen Q, Zhang X, Zhang B, Zhuo X, et al (2013) PCM1 recruits Plk1 to the pericentriolar matrix to promote primary cilia disassembly before mitotic entry. J Cell Sci 126: 1355–1365. 23345402 10.1242/jcs.114918

[28] Yen HJ, Tayeh MK, Mullins RF, Stone EM, Sheffield VC, et al (2006) Bardet-Biedl syndrome genes are important in retrograde intracellular trafficking and Kupffer's vesicle cilia function. Hum Mol Genet 15: 667–677. 16399798 10.1093/hmg/ddi468

[29] Tayeh MK, Yen HJ, Beck JS, Searby CC, Westfall TA, et al (2008) Genetic interaction between Bardet-Biedl syndrome genes and implications for limb patterning. Hum Mol Genet 17: 1956–1967. 18381349 10.1093/hmg/ddn093 PMC2900902

[30] Seo S, Baye LM, Schulz NP, Beck JS, Zhang Q, et al (2010) BBS6, BBS10, and BBS12 form a complex with CCT/TRiC family chaperonins and mediate BBSome assembly. Proc Natl Acad Sci U S A 107: 1488–1493. 20080638 10.1073/pnas.0910268107 PMC2824390

[31] Pretorius PR, Baye LM, Nishimura DY, Searby CC, Bugge K, et al (2010) Identification and functional analysis of the vision-specific BBS3 (ARL6) long isoform. PLoS Genet 6: e1000884. 20333246 10.1371/journal.pgen.1000884 PMC2841623

[32] Wilkinson CJ, Carl M, Harris WA (2009) Cep70 and Cep131 contribute to ciliogenesis in zebrafish embryos. BMC Cell Biol 10: 17. 19254375 10.1186/1471-2121-10-17 PMC2662791

[33] Baye LM, Patrinostro X, Swaminathan S, Beck JS, Zhang Y, et al (2011) The N-terminal region of centrosomal protein 290 (CEP290) restores vision in a zebrafish model of human blindness. Hum Mol Genet 20: 1467–1477. 21257638 10.1093/hmg/ddr025 PMC3063982

[34] Pretorius PR, Aldahmesh MA, Alkuraya FS, Sheffield VC, Slusarski DC (2011) Functional analysis of BBS3 A89V that results in non-syndromic retinal degeneration. Hum Mol Genet 20: 1625–1632. 21282186 10.1093/hmg/ddr039 PMC3063988

[35] Easter SS Jr, Nicola GN (1996) The development of vision in the zebrafish (Danio rerio). Dev Biol 180: 646–663. 8954734 10.1006/dbio.1996.0335

[36] Sanger WT (1998) Ctalogue of somatic mutations in cancer. In: Institue WTS, editor. Ctalogue of somatic mutations in cancer. Hinxton, UK: Wellcome Trust Sanger Institute.

[37] Ma L, Jarman AP (2011) Dilatory is a Drosophila protein related to AZI1 (CEP131) that is located at the ciliary base and required for cilium formation. J Cell Sci 124: 2622–2630. 21750193 10.1242/jcs.084798 PMC3138703

[38] Marion V, Stutzmann F, Gerard M, De Melo C, Schaefer E, et al (2012) Exome sequencing identifies mutations in LZTFL1, a BBSome and smoothened trafficking regulator, in a family with Bardet–Biedl syndrome with situs inversus and insertional polydactyly. J Med Genet 49: 317–321. 22510444 10.1136/jmedgenet-2012-100737

[39] Nachury MV (2008) Tandem affinity purification of the BBSome, a critical regulator of Rab8 in ciliogenesis. Methods Enzymol 439: 501–513. 18374185 10.1016/S0076-6879(07)00434-X

