# Supplementary figures and images for: The Centriolar Satellite Protein AZI1 Interacts with BBS4 and Regulates Ciliary Trafficking of the BBSome
Source: PLoS Genet. 2014 Feb 13;10(2):e1004083. doi: 10.1371/journal.pgen.1004083 (PMC3923683; doi:10.1371/journal.pgen.1004083)

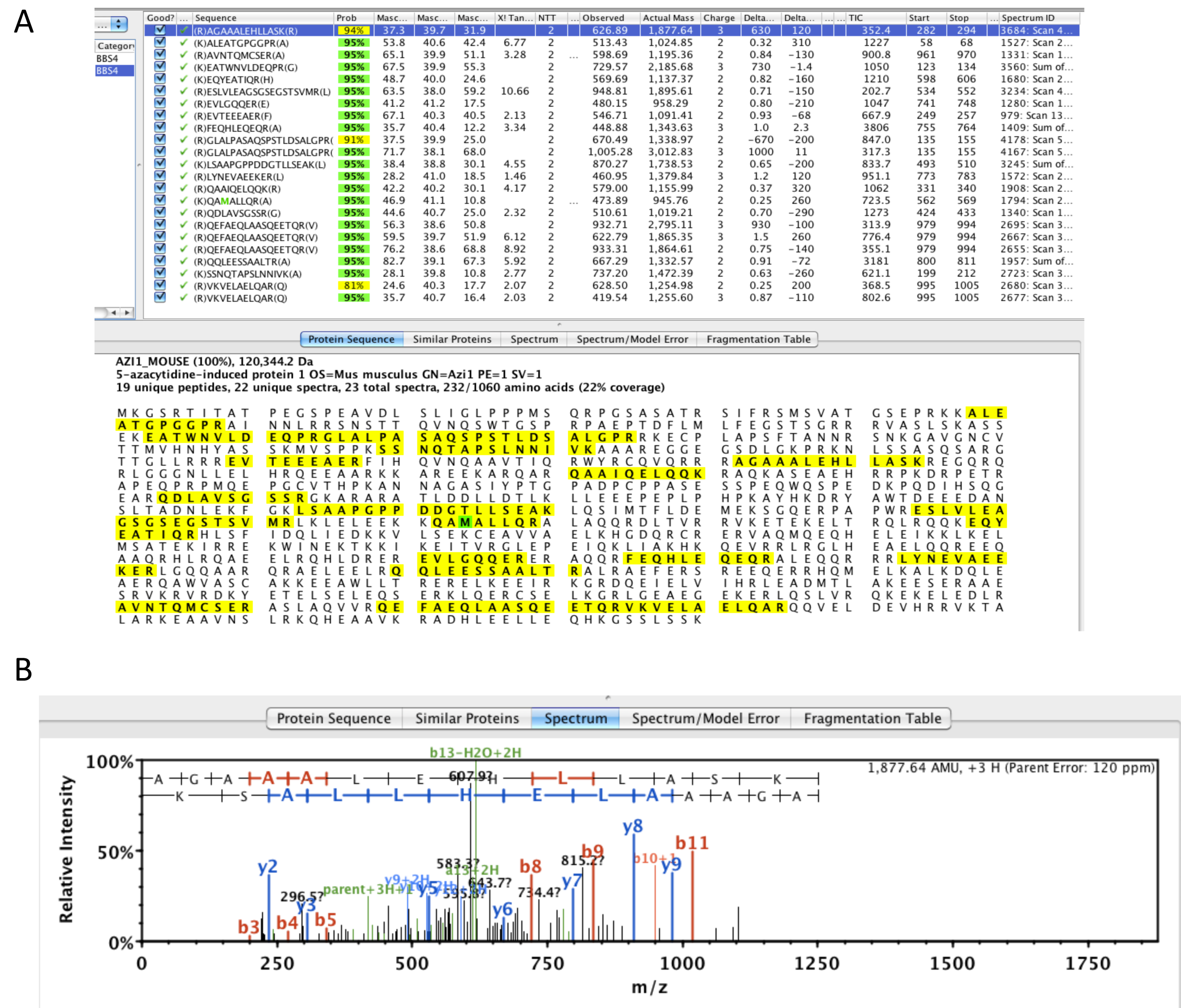

Supplement: Figure S1 — Identification of AZI1. Screen-shot from scaffold3 software showing the unique peptides identified from the 120 KD band. The peptide coverage (A) and mass spectrum plot (B) shows identification of the AZI1. (TIF) [file pgen.1004083.s001.tif]

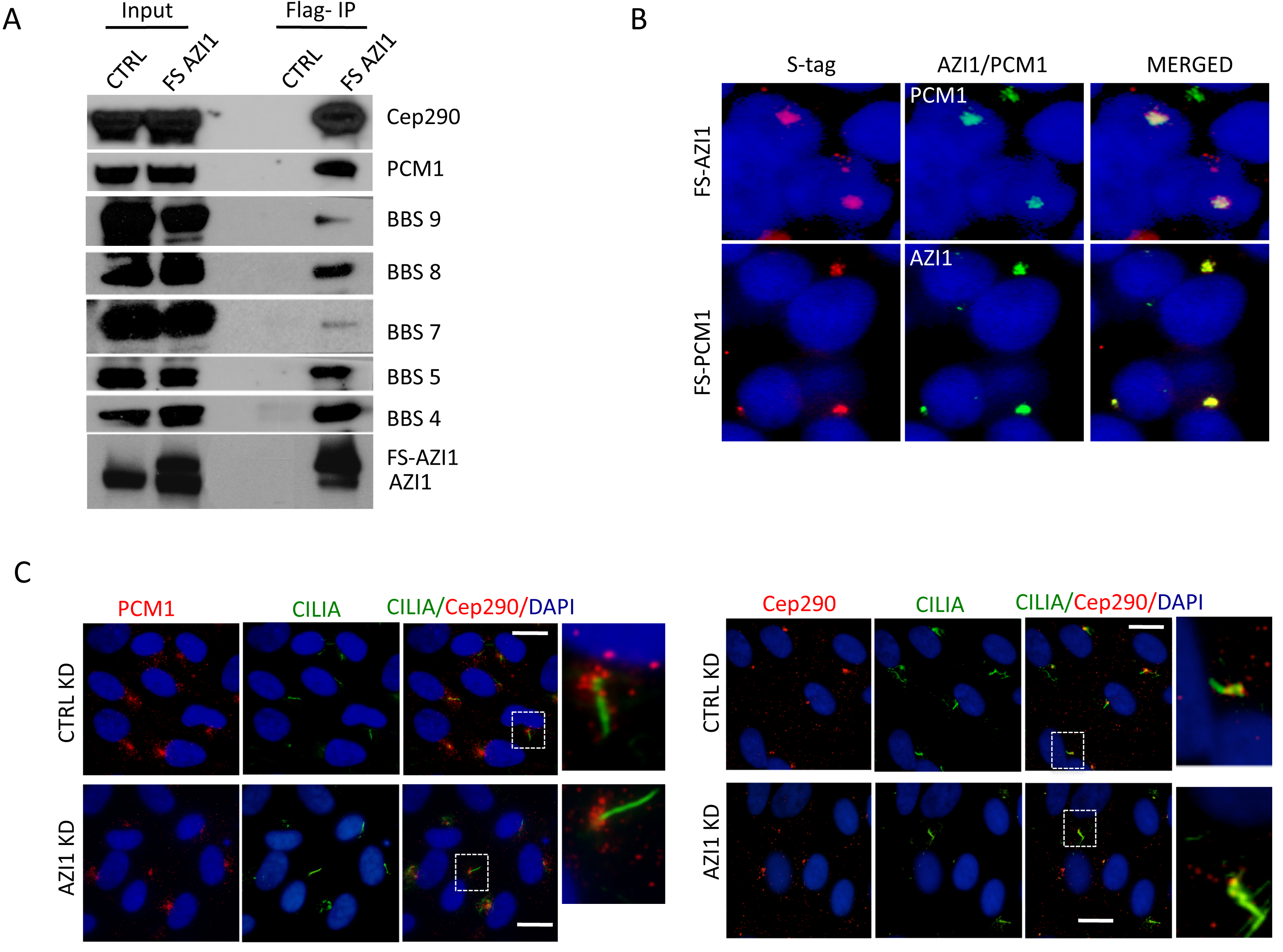

Supplement: Figure S2 — AZI1 interacts with the BBSome complex and satellite proteins. A) FLAG and S tagged AZI1 were stably expressed in 293T cells, and Co-IP was performed using FLAG agarose beads. Input (total lysate) and the final precipitate were run on SDS-PAGE and Western blotted using antibody against CEP290, PCM1, BBS4, BBS5, BBS7, BBS8, and BBS9. Centriolar proteins as well as the other BBSome subunits along with FS-AZI1, and endogenous AZI1 were precipitated by AZI1. B) AZI1 co-localizes with PCM1 in non-ciliated cells. 293T cells stably expressing FS-PCM1 or FS-AZI1 were stained with antibody against S-tag (red) and PCM1 or AZI1 (green). C) siRNA knockdown of AZI1 has no effect on centriolar satellite localization of PCM1 or Cep290 (red). Cilia are stained with acetylated α-tubulin. Nuclei are stained blue with DAPI. (TIF) [file pgen.1004083.s002.tif]

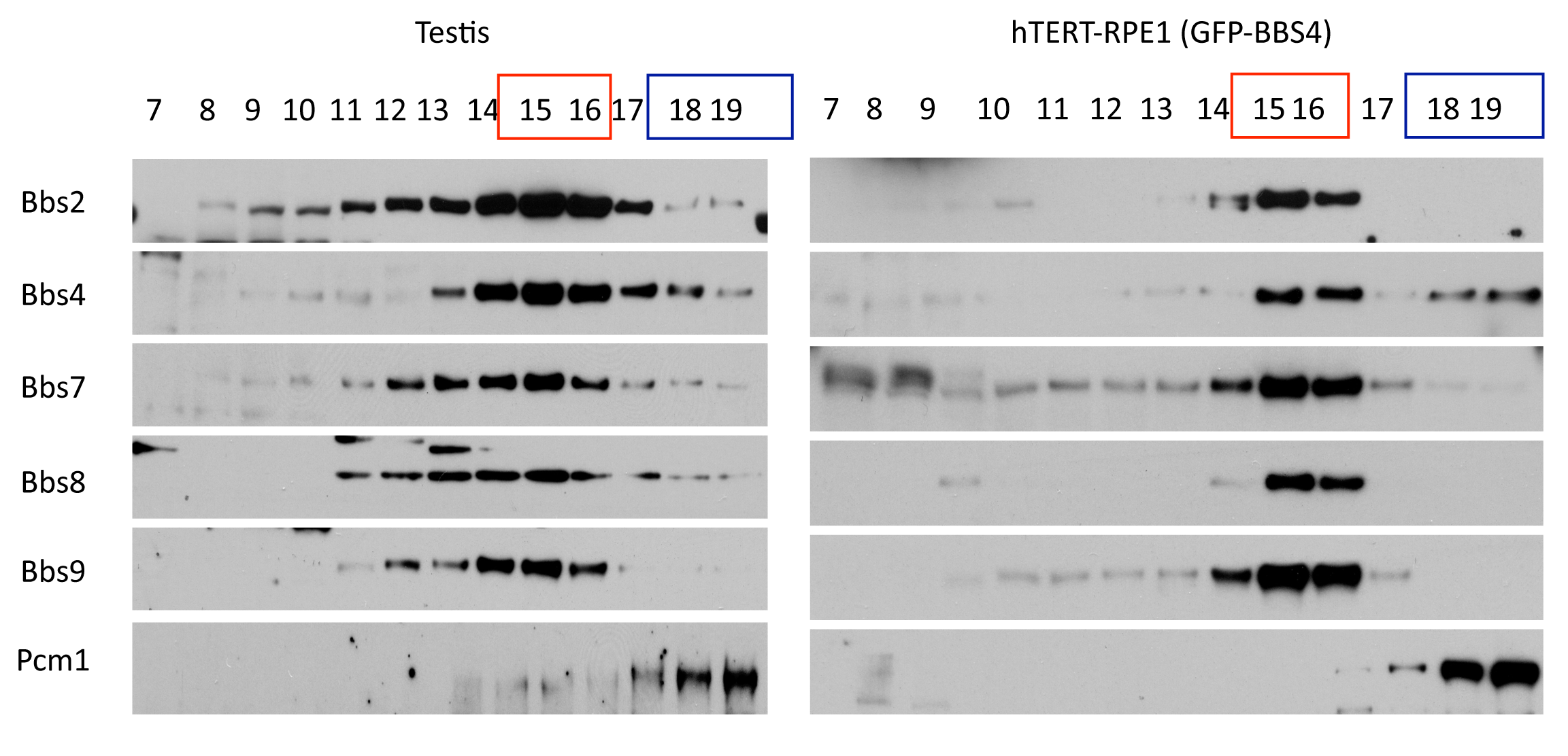

Supplement: Figure S3 — BBS4 is part of a centriolar satellite complex. Sucrose gradient (5%–25%) centrifugation followed by Western blotting was performed on protein lysates from testis and LAP-BBS4 stable cells. Two pools of BBS4 are seen in GFP-BBS4 cells. Although BBS4 extends towards the heaver fraction where PCM1 is seen, no evident separate pools of BBS4 was seen in the testis. (TIF) [file pgen.1004083.s003.tif]

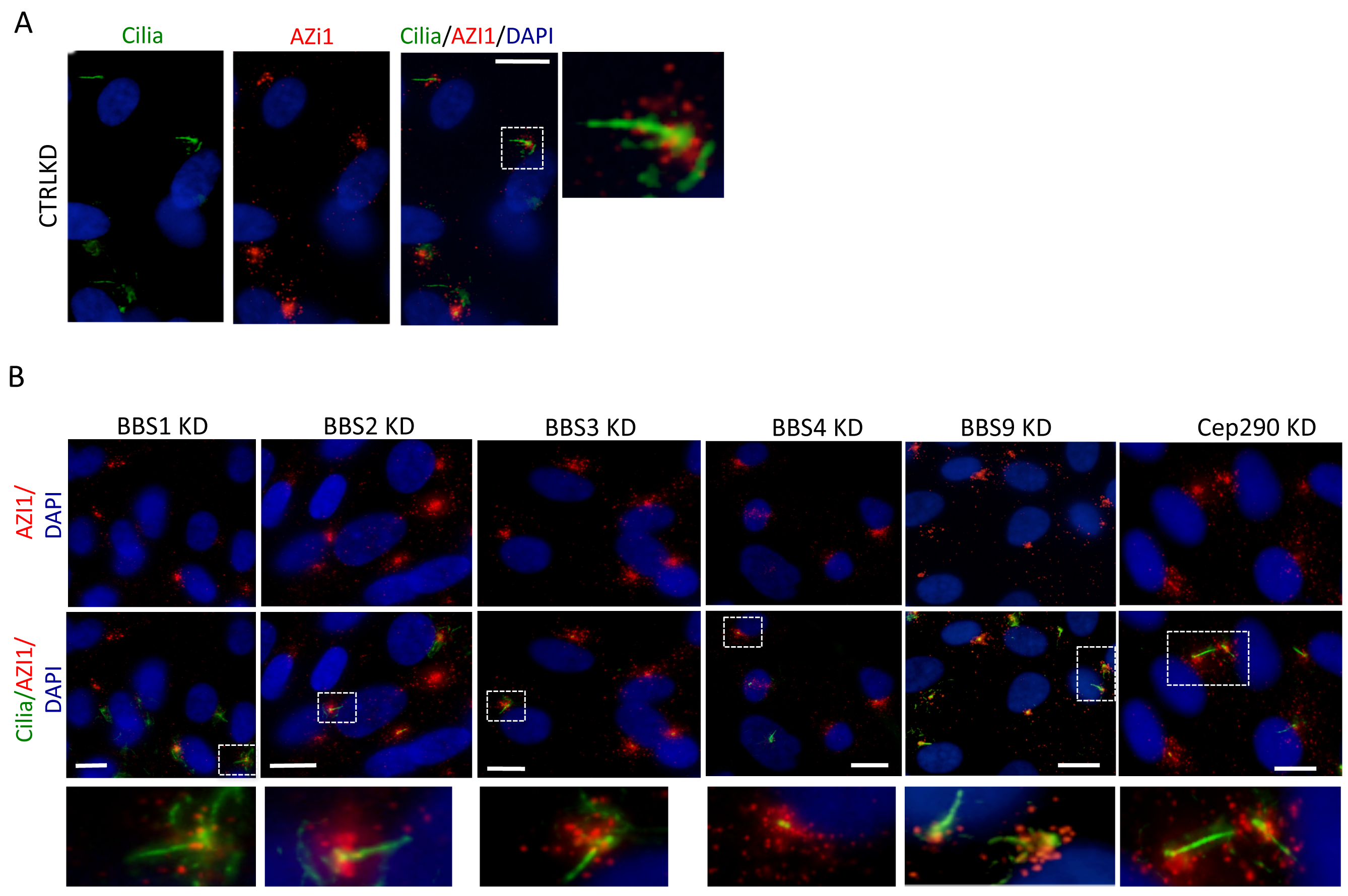

Supplement: Figure S4 — Localization of AZI1 is not affected by BBS knockdown. RPE-1 cells were transfected with siRNA against different BBS genes and the localization of AZI1 was analyzed. A) Localization of AZI1 upon control knockdown. B) AZI1 localization upon different BBSome proteins and Cep290 knockdown. No significant difference in the centriolar satellite localization of AZI1 is detected upon BBS proteins or Cep290 knockdown. Cilia and basal body are stained with acetylated α-tubulin and γ-tubulin respectively; red staining is AZI1, and nuclei are stained (blue) with DAPI. (TIF) [file pgen.1004083.s004.tif]

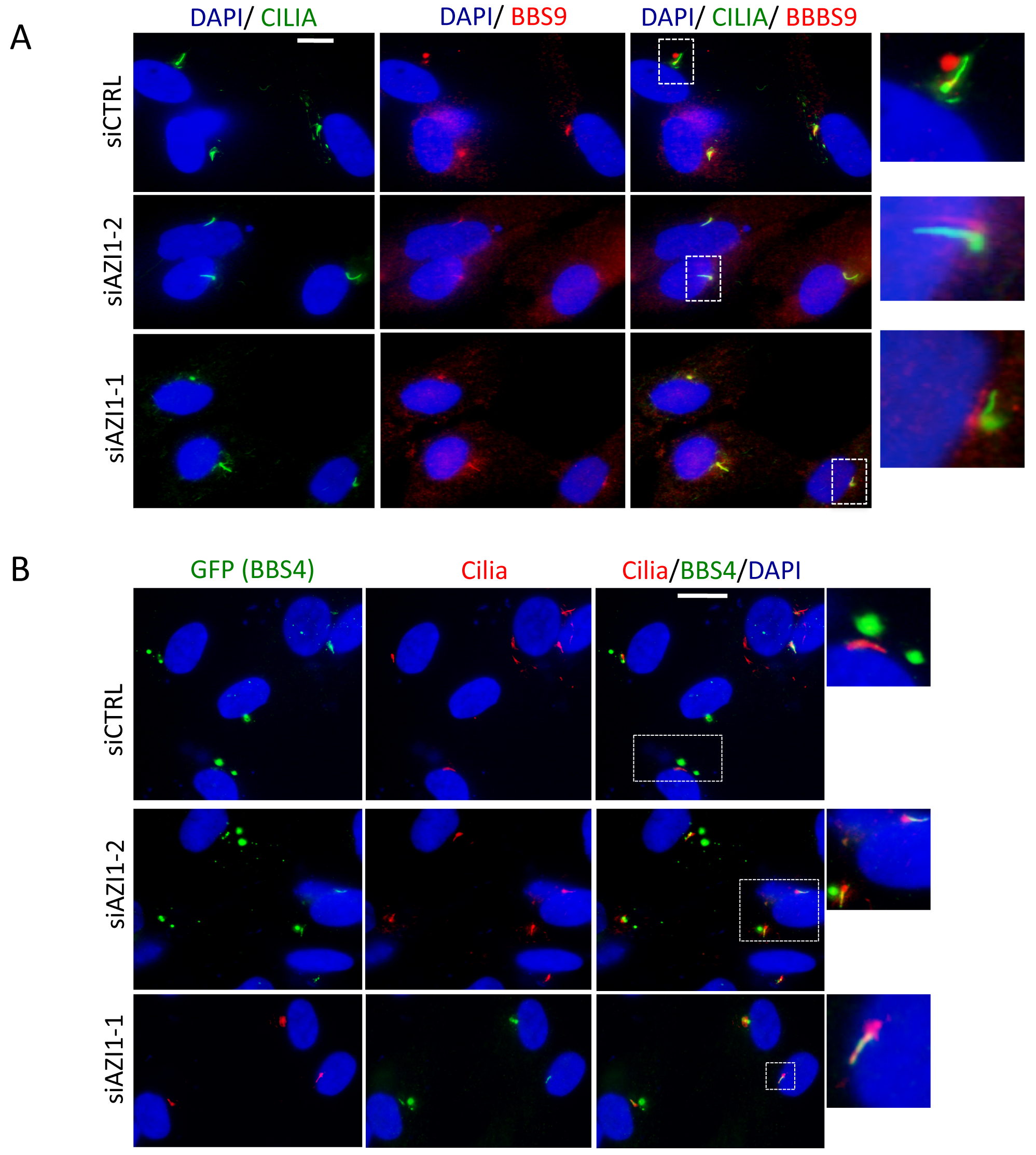

Supplement: Figure S5 — AZI1 knockdown effects the ciliary localization of the BBSome. Ciliary localization of BBS9 (red) (A) or GFP-BBS4 (green) (B) is increased upon AZI1 knockdown by various AZI1 siRNAs. Cilia (green) in figure A is slightly shifted to show BBS9 localizes in cilia. Cilia are stained with acetylated α-tubulin. Nuclei are stained blue with DAPI. (TIF) [file pgen.1004083.s005.tif]

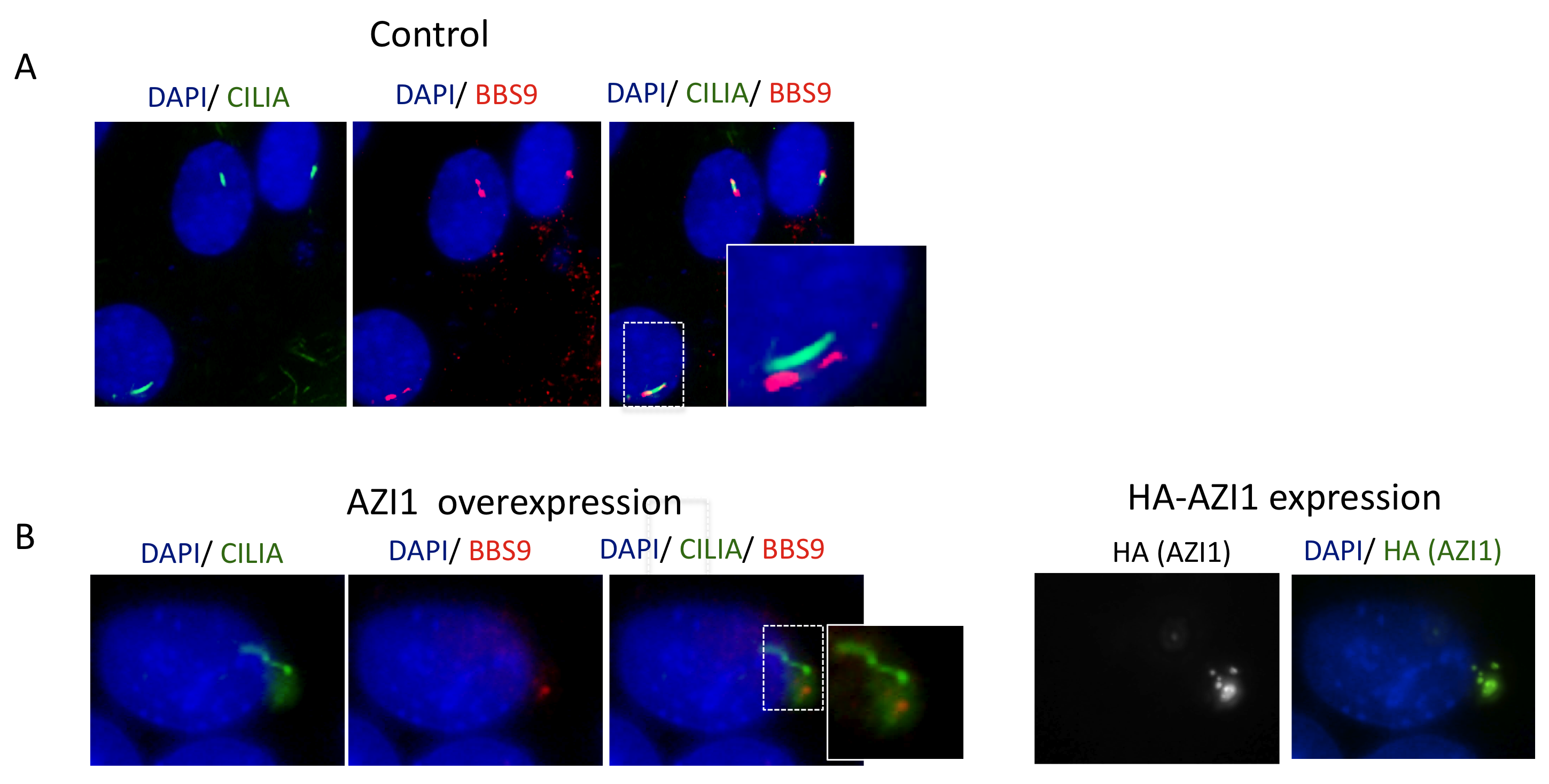

Supplement: Figure S6 — Overexpression of AZI1 reduces ciliary localization of BBS9. Cells were transfected with 0.25 µg of HA-AZI1 construct, and ciliary localization of BBS9 was analyzed. A) In control cells, ciliary BBS9 (red) is apparent, but no ciliary localization of BBS 9 in the AZI1 overexpressed cells was observed B). Effective transfection and HA-AZI1 expression is indicated in the last two images; HA staining (pseudo colored green) is also included for better comparison. Cilia are stained with acetylated α-tubulin and nuclei are stained with DAPI. (TIF) [file pgen.1004083.s006.tif]

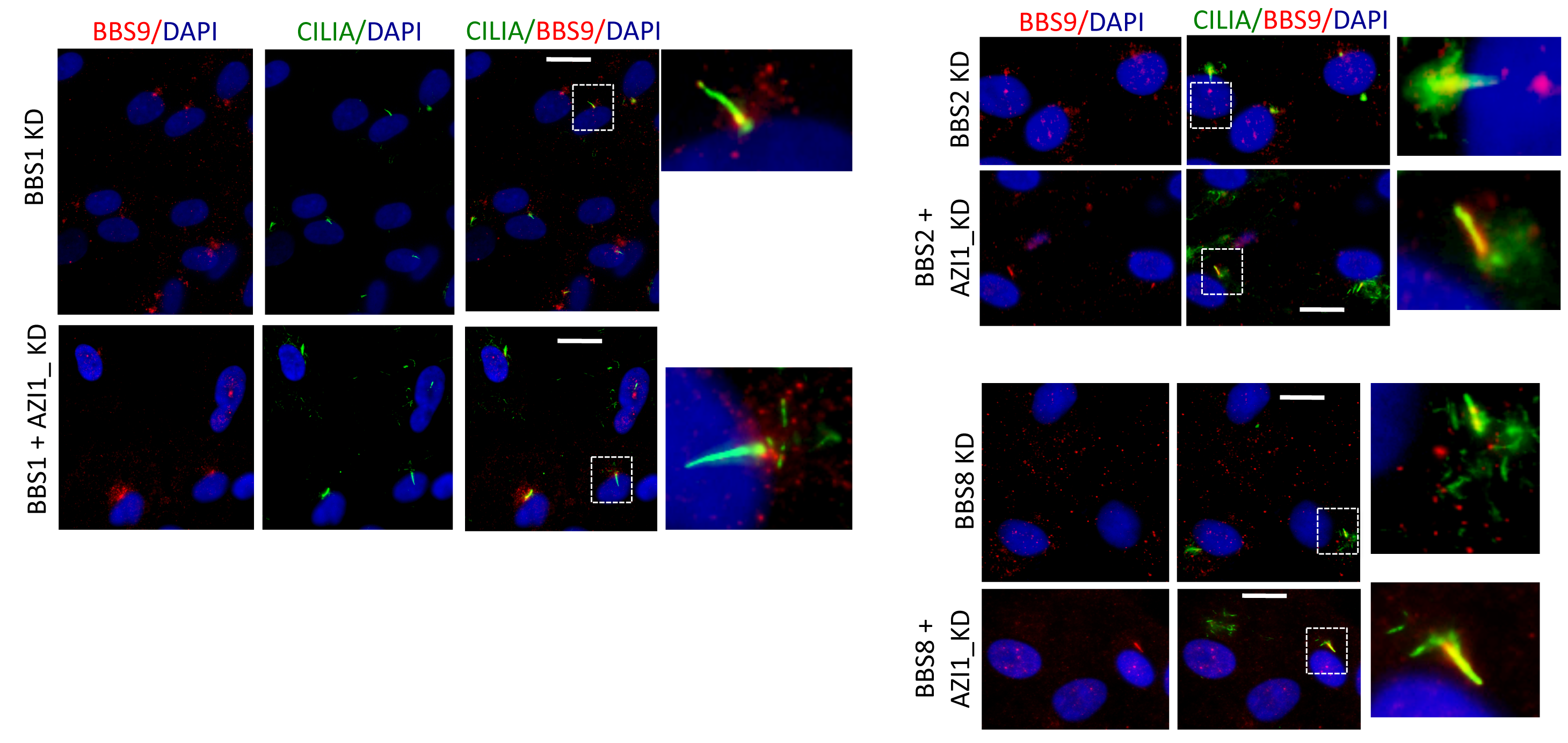

Supplement: Figure S7 — Localization of BBS9 upon knockdown of BBS proteins and AZI1. BBS proteins (BBS1, BBS2, and BBS8) are depleted in RPE-1 cells and loss of BBS9 (red) localization in cilia (green) is apparent (first row each panel). Knockdown of AZI1 in cells depleted of BBS protein rescues ciliary localization of BBS9 except in BBS1 depleted cells (second row each panel). (TIF) [file pgen.1004083.s007.tif]
